# *Denovo* variants in *POGZ* and *YY1* genes: The novel mega players for neurodevelopmental syndromes in two unrelated consanguineous families

**DOI:** 10.1371/journal.pone.0315597

**Published:** 2025-01-08

**Authors:** Behjat Ul Mudassir, Mujaddid Mudassir, Jamal B. Williams, Zehra Agha

**Affiliations:** 1 Translational Genomics Laboratory, Department of Biosciences, COMSATS University, Islamabad, Pakistan; 2 Rawalpindi Institute of Cardiology, Rawalpindi, Pakistan; 3 Department of Psychiatry, Jacobs School of Medicine and Biomedical Sciences, State University of New York at Buffalo, Buffalo, NY, United States of America; Shaheed Rajaei Hospital: Rajaie Cardiovascular Medical and Research Center, ISLAMIC REPUBLIC OF IRAN

## Abstract

Novel *denovo* variants of exome sequences are major cause of pathogenic neurodevelopmental disorders with a dominant genetic mechanism that emphasize their heterogeneity and complex phenotypes. White Sutton syndrome and Gabriele-de-Vries syndrome are congenital neuro-impairments with overlap of severe intellectual disability, microcephaly, convulsions, seizures, delayed development, dysmorphism of faces, retinal diseases, movement disorders and autistic traits. *POGZ* gene codes for pogo transposable element-derived zinc-finger protein and *YY1* gene regulates transcription, chromatin, and RNA-binding proteins that have been associated with White Sutton and Gabriele-de-Vries syndromes, in recent data. We present probands of two unrelated consanguineous families with complicated, unexplained neurocognitive syndromic characteristics clinically undiagnosed. Objectives of the study were to identify altered genetics and protein characteristics underlying molecular pathological pathways in both the patients. Whole exome sequencing identifies novel, *denovo* missense variant NM_015100.4: c.776 C>T (p. Pro259Leu) in exons 19 of *POGZ* gene and non-frameshift variant NM_003403.5: c.141_143delGGA (p. Glu47del) in exon 1 of *YY1* gene for White Sutton syndrome in eight years five-month-old girl and Gabriele-de-Vries syndrome in seven years eight months old boy residing in Rawalpindi and Chakwal districts of Punjab, Pakistan respectively. Protein modelling for identified variants predicts size and conformation modifications in mutated amino acid residues that lead to damaging effects in the conserved domains expressed as neurological pathophysiology. The present study widens the diversely ethnic and highly inbred gene pool of Punjab, Pakistan population for spontaneously originated deleterious mutations and contributes to the continuously expanding phenotypic canvas. Molecular genetic identification and personalized diagnosis for the patients suffering from complicated neurodevelopmental phenotypes, for better care, management of day-to-day activities and prolonged life span are the utmost hopes.

## Introduction

Human beings receive one half of their genetic information from father and the other half from their mother [1]. A tiny proportion of alterations, known as *de novo* variations, are not found within either parent’s genome [2]. These can be freshly appeared during gamete production or occur during early embryonic development, making the offspring’s genome distinct from the parent [3]. Denovo variations can be as little as a single nucleotide variant or as big as massive deletions, duplications, or rearrangements in the genes due to faulty DNA replication or recombination, not rectified by proofreading [4]. Advancements in personal genome analysis and large cohort studies suggest a diverse categorization in the molecular and phenotypic basis of neurodevelopmental disorders (NDDs) that affect development of the nervous system to disrupt its proper functioning [5] and includes neuro cognitive characteristics of intellectual impairment [6], seizures [7], microcephalic [8] and ophthalmic manifestations [9], dysmorphic faces [10], with neurobehavioral features [11] such as autism, repetitive and hyperactivity disorders which are 1% to 3% prevalent collectively across the globe usually overlapping in one individual [12]. Recently, the identification of *denovo* variants in neurodevelopmental disorders afflicted patients has got the substantial genetic etiology [13] related to neurocognitive domains which is required to be investigated further [14]. More than one thousand genes were identified with *denovo* mutations that affect neurodevelopmental spectrum of disorders [15] causing pathological implications in synaptic function domains by missense variants in thirty five genes and recurrent alterations in thirty six genes [16]. The common neurodevelopmental phenotypes including intellectual disability, epilepsy and autism profoundly co-occurring because of novel and recurrent disruptions in *ARID1B*, *ANKRD11*, *KMT2A*, *ADNP*, *DDX3X*, *SYNGAP1*, *ASXL3*, *DYRK1A*, *SCN2A*, *SETD5*, *CTNNB1*, *POGZ*, *MED13L*, *CHD8*, *CHD2*, *EBF3*, *DHX30*, *EP300*, *KAT6B*, *MECP2*, *AHDC1*, *FOXP1*, *TCF4*, *WDR45*, *GATAD2B*, *KAT6A*, *SHANK3*, *TCF20*, *ACTL6B*, *ALG13*, *CDK13*, *COL4A3BP*, *YY1*, *GABBR2*, *GRIN2B*, *KCNH1*, *KCNQ2*, *KIFSC*, *PACS1*, *PACS2*, *PCGF2*, *PPP2R1A*, *PPP3CA*, *PP2RSD*, *SMAD4* and many other genetic pathways due to dominant denovo truncations and approximately six to nine percent of all neurocognitive diagnosis [17]. Identification and ranking of NDDs risk genes is critical for understanding the underlying physiological processes that undergo disruption in NDDs. Prior studies have uncovered various monogenic as well as the multifactorial and polygenic variants for the majority of NDDs [18].

*POGZ* or White-Sutton syndrome [19] shows significant predominance of cognitive impairment delayed development, concomitant autistic traits, auditory pathologies and commonalities at the molecular level [20] due to pathological impacts on chromatin regulators and transcription factors that participate in gene repression, mitotic progression, and DNA repair [21]. White-Sutton syndrome is a rare genetic condition caused by denovo heterozygous pathogenic *POGZ* variants. Over 120 individuals have been documented globally so far [22], although phenotypic characterization and natural history data are still lacking [23, 24]. Pogo transposable element-derived protein with zinc finger domain is a Heterochromatin Protein 1α-binding protein has zinc finger domains, centromere protein B like DNA binding motif, Helix-turn-Helix, HP1 binding zinc finger binding motif, that destabilizes the HP1α and chromatin connection to dissociates Aurora B kinase from the chromosomal arm during meiosis and mitosis. Malfunctioning of POGZ leads to abnormal cell differentiation and inhibition of growth that develops neurodevelopmental disorders and microcephalic phenotypes [25]. POGZ additionally associates with the SP1 transcription element and the chromodomain helicase DNA-binding protein 4, indicating that it operates as a chromatin organizer and is thus necessary for appropriate mitotic progress, particularly during cortex differentiation [26]. The POGZ-related diseases have a variety of identified genomic variations including missense, nonsense, and frameshift as well as deleterious variants appear to be associated with behavioral anomalies, intellectual disability, hearing, vision, gastrointestinal or urinary tract malformations. However, no obvious genotype-phenotype connection has been established. There is, yet no approved diagnostic standards for White-Sutton syndrome, and no distinguished phenotype for this condition has been discovered in cases previously reported [27]. Likewise, this is heterogeneous at the levels of phenotype as well as genotype and the only way to properly diagnose this condition is to look for alterations in the POGZ gene, which may result in lack or malfunction of POGZ protein and are linked to a number of neurodevelopmental manifestations [28].

Alterations in the *YY1* gene, causes a unique autosomal dominant neurodevelopmental condition firstly reported by Gabriele et al. (2017). Gabriele-de Vries syndrome [29] is characterized by craniofacial dysmorphism including broad forehead ridge, arched bushy eyebrows, broad nose, low-set ears, with low birth weight, feeding difficulties, varying degrees of delayed development, intellectual disability, speech and language delay, attention deficit/hyperactivity disorder, autistic behavior, schizophrenia, anxiety disorder, ataxia, and seizures [29]. In literature, around 13 cases reported so far for this syndrome [30] *YY1* gene with its five exons that code for a four hundred and fourteen amino acid long zinc finger protein transcription factor, on chromosome fourteen long arm (32.2 position). The *YY1* gene mutation analyzed in variety of patients include missense, frameshift, nonsense variations and deletions in initiation codon, exon 4 and 5, suggesting that these exons may be *YY1* mutation hotspots [30–32]. The fundamental genetic driving force behind the disorder was haploinsufficiency of the *YY1* gene. Convergent evidence implies that *YY1* has a direct role in neuronal development and activity. *YY1* gene haploinsufficiency can result in substantial loss of H3K28 acetylation, rendering the H3K27 substrate accessible for PrC2-mediated methylation and thereby limiting gene expression [33]. Gabriele-de Vries syndrome, caused by a *YY1* gene mutation, shows variety of clinical symptoms [34], varied degrees of severity, and no precise diagnostic procedure [32]. An early detection and screening of genome of the patients by whole-exome sequencing can be feasible for identification of strongly suspected genetic disorder to confirm the diagnosis, which can aid with clinical therapy and genetic counselling to better estimate patients’ prognosis [35]. Although there is no particular treatment for this condition, symptomatic treatment and extensive rehabilitation therapy can enhance children’s quality of life [36]. For this condition, neurodevelopmental testing is required, particularly early assessment of gross and fine motor function and language. Early identification can lead to earlier intervention in rehab training [37]. Here, we report for the first time in the population of Punjab province, two unrelated highly consanguineous families with *POGZ* and *YY1* gene denovo mutations identified by WES in two probands, resulting phenotypes of White Sutton syndrome and Gabriele-de-Vries syndrome respectively. This study has revealed the mechanism of *denovo* genetic alterations in *POGZ* and *YY1* genes that lead to malfunctioned expression of truncated proteins and resulting disease phenotypes in probands. Our study has impacted the otherwise unexplained neurocognitive spectrum of syndromes caused by *POGZ* and *YY1*. We expect that our study will add to the genotypic and phenotypic manifestations in rare and complex neurological syndromes caused by *denovo* gene mutations. Furthermore, this pioneer discovery will open the research for White Sutton and Gabriele-de-Vries syndromic patients in Pakistan where tertiary health care for genetic disorders is scarce, and human genetic research groups are working in collaboration with medical departments and global consortia to cope up with the shortfall of facilities.

## Material and methods

### 1. Proband recruitment criteria, clinical diagnosis, ethical approval, and consent from families

Field trips were organised from 20^th^ July 2021 to 30^th^ August 2022 to explore the neurologic syndromic patients in various areas of highly populated Punjab Province, by Professors and research scholars of Translational Genomics Research Group, COMSATS University, Islamabad. The aim was to provide genetic testing facilities and counselling to the deprived and undiagnosed patients suffering from neurodevelopmental disorders. Two unrelated highly inbred families, ES-4, and ES-10, with probands showing undiagnosed rare phenotypes of neurocognitive syndromes were recruited from Rawalpindi and Chakwal areas respectively. This study was approved by ethical review board of Biosciences Department, COMSATS University, Islamabad wide notification CIIT/Bio/ERB/17/50 dated 09-10-2017. The standards and norms of the research were in accordance with the **Declaration of Helsinki** ethical guidelines. Parents of the probands were informed about the study in detail by distribution of questionnaires and written consent was obtained to collect blood samples for whole exome sequencing and publication of the data.

Proband of family ES-4 was eight years five-month-old girl residing in Rawalpindi, with comorbid milestones of microcephaly, severe intellectual disability (IQ<30), craniofacial characteristics that includes expanded forehead ridge, widely spaced highly arched eyes with bushy eyebrows, broader nose, low-set ears, microdontic, impaired neuro-cognition with slower than normal cognitive and motor development, convulsions, as well as delayed speech and social skills. She had severe ASD manifestation such as repetitive behaviors, aggression, resistance to adapt to the changes in the environment, while ophthalmic impairment with bilateral cataract was also observed clinically. Height was short 40 in (-5.36 Height-to-age Z-score) with obese body 90 lb. (1.87 Weight-to-age Z-score). MRI findings as reported by the radiologist in the proband ES-4 were hyperintensities in frontal region and reduction in volume of white matter in midbrain. She retained her set of milk teeth, frequent gastric disturbances, and episodes of tachycardia were observed. Proband ES-4 had anxiety in mood with depressive periods, sleep disturbances and recurrent urinary infections. Initial clinical findings by general physician diagnosed her as suspect of White Sutton syndrome but her exact phenotype was not confirmed based on clinical examination only and genetics was required to ascertain the genomic variation.

Proband of Family ES-10 was seven years eight months old boy residing in village area of Chakwal district, Punjab. He visited a basic health care unit near his home for symptoms of intellectual impairment (IQ<25), facial dysmorphic characteristics with frontal bossing, expanded nose, deep eyes, low and expanded ears, congenital dental anomalies, bilateral retinal degeneration, psychiatric manifestations in mood, movement disorder including ataxia, seizures, and hypotonia. He was short in height 38 in (-5.57 Height-to-age Z-score) and low weight 30 lb. (-6 Weight-to-age Z-score) Brain MRI of proband ES-10 as reported in the radiologist observations was abnormal with underdeveloped corpus callosum (partial agenesis) and reduced white matter in cerebrum. Clinical examination by general physician diagnosed the proband as suspect of Gabrielle-de-Vries syndrome while the phenotype identification required proper genetic assessment as well.

Both the probands of these unrelated families were referred to Benazir Bhutto Hospital neurologists and psychiatrists for the confirmation of initial clinical findings. Detailed clinical examination at neurology, psychiatry, cardiology, pathology, ophthalmology and orthopedic clinics, Proband ES-4 was diagnosed as White Sutton syndrome phenotype and proband ES-10 was diagnosed as Gabriele-de-Vries syndrome phenotype. The observed phenotypic manifestations in the probands ES-4 and ES-10 were comparable with the classical milestones of the White Sutton and Gabriele-de-Vries syndrome respectively. Neurologic consultants suggested the families to get genetics, to confirm clinical diagnosis and pathogenic genomic variant. Translational Genomics research group in collaboration with team of consultants, started genetic identification procedure.

### 2. Pedigree assessment, blood sample collection and gDNA extraction in the lab

Pedigrees of Families ES-4 and ES-10 were kept up to five generations and history of any previous neurologic impairment was traced by interviewing the parents. Pedigree ES-4 didn’t show any history of neurological disorder in the family except the proband III-1 while pedigree ES-10 shows history of diagnosed neurological illnesses in the previous and current generations as intellectual disability (III-3), Dyscephaly (IV-1), IDM (IV-3), ASD (IV-9) in addition to proband (IV-7). Clinical symptoms were recorded and weight-for-Height/BMI Z-scores were calculated for both the probands to investigate and validate the physical characteristics. 2.5 ml peripheral blood samples from probands were drawn, their unaffected biological parents and siblings were collected in EDTA tubes and processed at translational genomics lab for DNA extraction by phenol-chloroform method and preserved at -20°C for further use.

### 3. Whole exome Sequencing, VCF collection, variant filtration and priotization

DNA samples were sent for whole exome sequencing at Macrogen Inc. Korea by Illumina Nova Seq 6000 sequencer. Received data VCF files for both the probands (ES-4 and ES-10) were uploaded to Franklin Genoox online software and resultant variants were subjected to bioinformatics analysis by *in silico* prediction tools Polyphon2, SIFT, Mutation Assessor, Mutation Taster, DANN and FATHMM. Evaluation of the variants was performed by the American College of Medical Genetics criteria for human genetics syndromic disorders. The frequency of the alleles was examined in control population databases that includes 1000 Genomes, gnomAD and ESP6500 for rare variant identification and pathogenicity detection. Those variants which have more than 0.01 minor allele frequency in any of human genome databases (1000 Genomes Project, Genome Aggregation Database, Exome Sequencing Project 6500, and database of single nucleotide polymorphisms were excluded from analysis. Resultant variants were filtered and prioritized by inhouse pipeline for identification of variants associated with proband ES-4 and ES-10 phenotypes.

## Results

### Demographical and phenotypic attributes for the family ES-4 and ES-10: (Tables 1 and 2)

**Fig 1:**
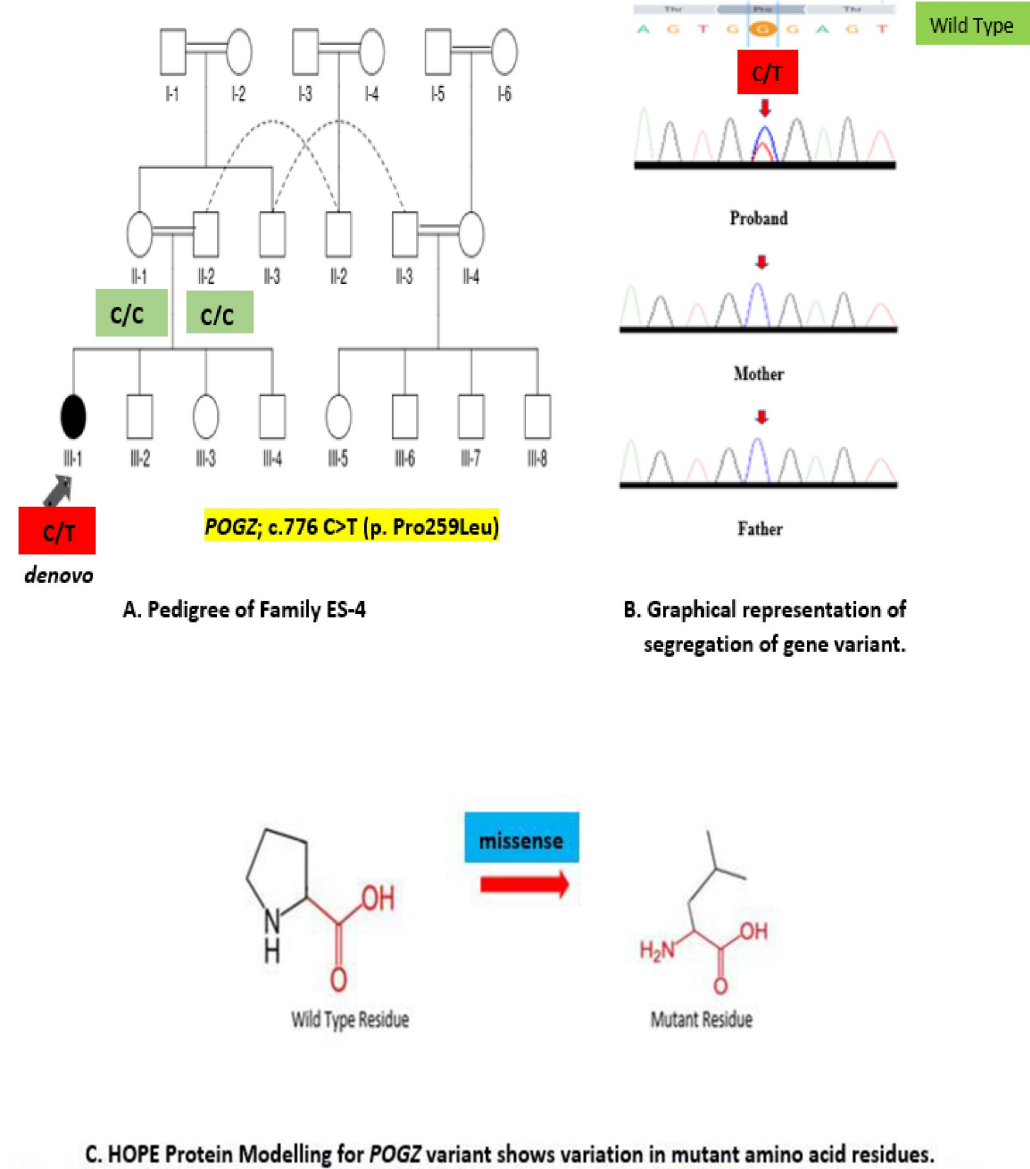
(A) Pedigree analysis of family ES-4 (proband III-1), (B) Segregation of POGZ novel, *denovo* variant in the family (C) Analysis of functional properties of mutant residue.

**Fig 2:**
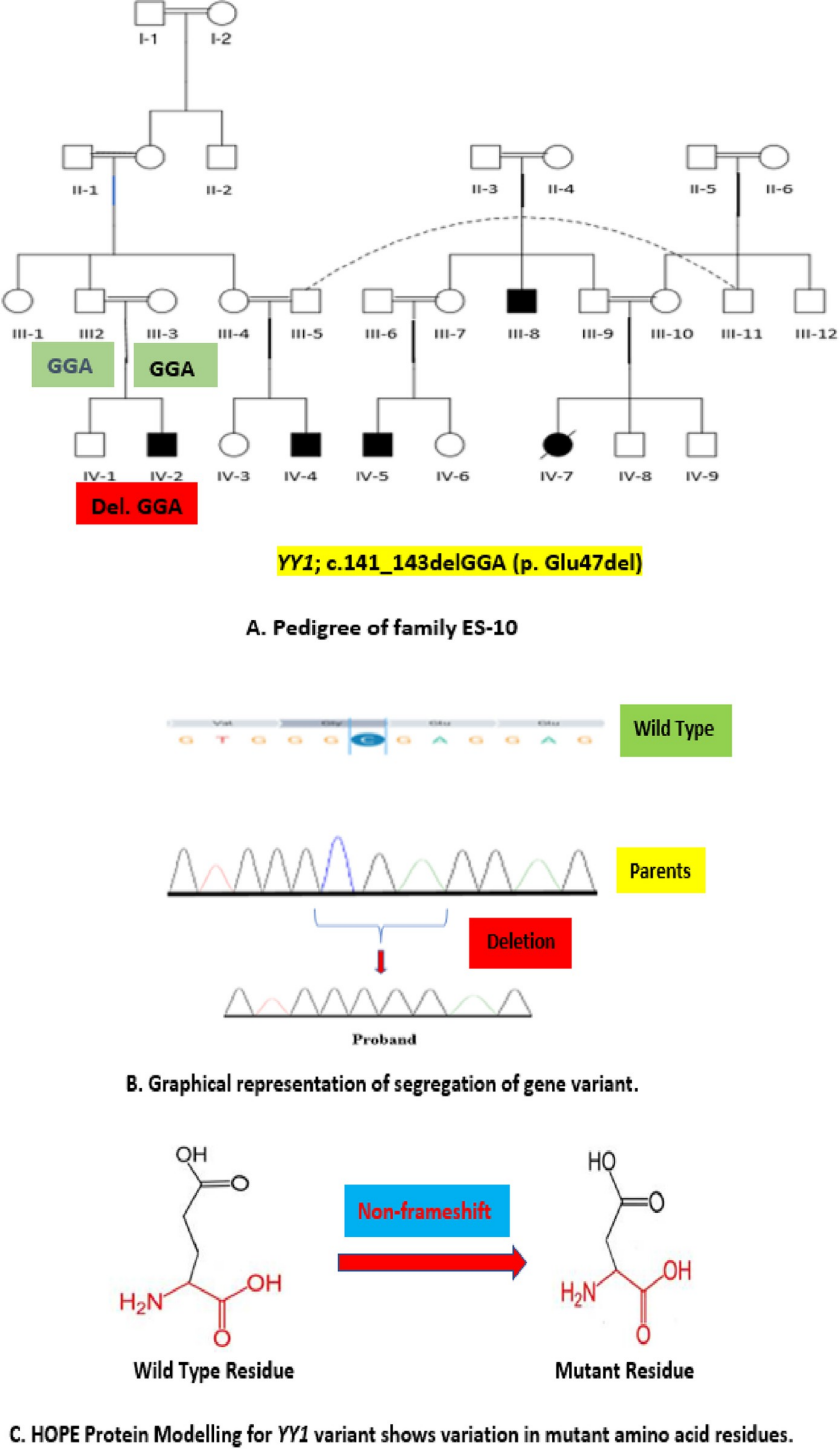
(A) Pedigree analysis of family ES-10 (proband IV-2), (B) Segregation of YY1 novel, *denovo* variant in the family (C) Analysis of functional properties of mutant residue.

**Fig 3:**
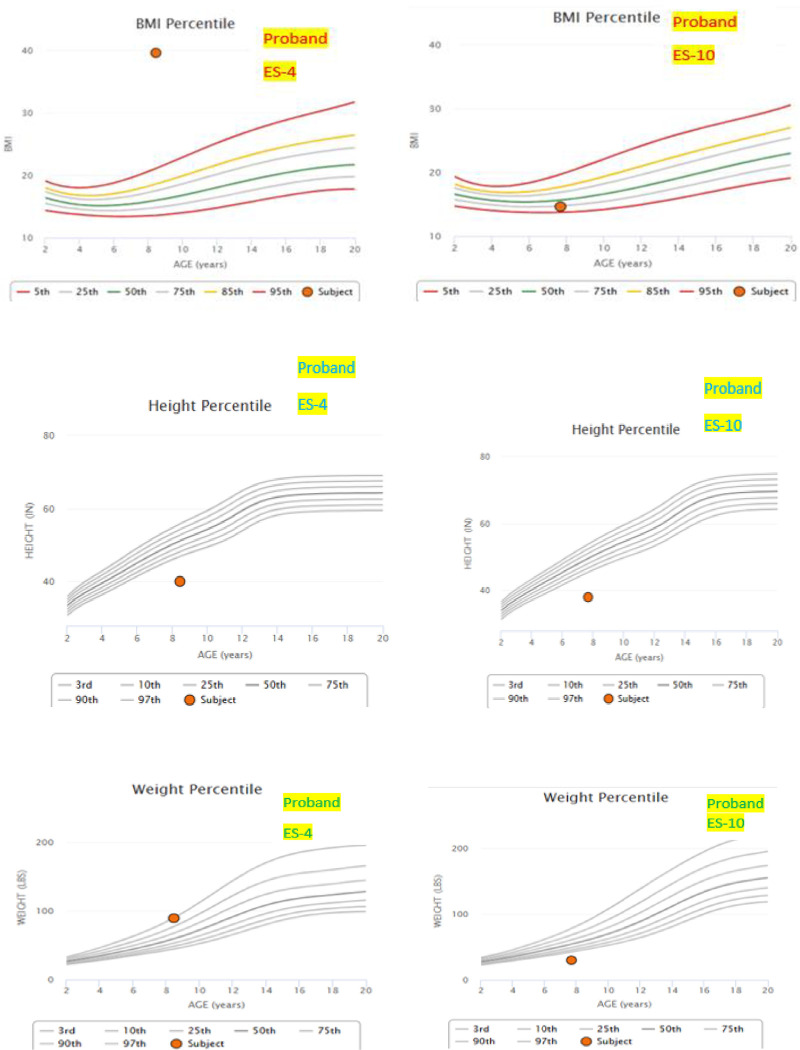
Height-to-age, Weight-to-age and BMI-to-age Z-Score/percentile analysis of the probands of the family ES-4 and ES-10 respectively.

- **Family ES-4** was multiplex family with consanguineous marriage history in several generations, but no report of any neurodevelopmental phenotype was provided by the parents during the pedigree assessment and analysis. Proband ES-4, a girl (III-1) was born to normal parents after first cousin marriage while all of her sibling were phenotypically normal. **(Fig 1)** The mother reported no complication during the pregnancy, and she had a normal delivery. Proband had congenital defects of neurodevelopment and neurological disorders including severe cognitive decline, microcephaly, seizures, autistic traits and mood disorders. Her developmental milestones revealed delayed and slow growth pattern, which were more evitable with increasing age.

- **Family ES-10** was highly inbred family with multiple consanguineous marriages and positive history of various neuro-cognitive manifestations in previous generations. Proband, a boy **(IV-2)** was born to normal parents after consanguineous marriage while his sibling was a phenotypically normal boy. **(Fig 2)** The mother reported that there was no history of complication during pregnancy and birth. Proband had intellectual disability, dysmorphism, delayed motor development, psychological manifestations and retinal degeneration.
- Height-to-age, Weight-to-age and BMI-to-age Z-Score/percentile analysis of the probands of the family ES-4 and ES-10 respectively shows that proband ES-4 is obese while ES-10 is weaker when compared to the median standard values of the same age group. The values of height Z-scores show both the probands shorter than the median standard values of same age group **(Fig 3)**.

**Table 1:** Phenotypic attributes of probands ES-4 and ES-10.

Milestones	Proband ES-4	Proband ES-10
**Microcephaly**	+	-
**Intellectual Disability**	IQ<30	IQ<25
**Epilepsy**	+	+
**ASD**	+	+
**Psychiatric behaviour**	+	+
**Facial Dysmophism**	+	+
**Dental anomalies**	+	+
**Anxiety**	+	+
**GIT**	+	+
**Auditory**	+	-
**Ophthalmic**	+	+
**Obesity**	+	-
**Hernia**	-	-
**Urinary**	+	-
**MRI**	+	+
**Cardiac**	+	-
**Sleep disturbance**	+	+
**Short Stature**	+	-

**Table 2:** Demographic attributes of the families ES-4 and ES-10.

Parameter	Family ES-4	Family ES-10
**Proband ID**	ES-4	ES-10
**Gender/Age**	F/8.5 years	M/7.8 years
**Age at onset**	By Birth	By Birth
**Diagnosis**	White Sutton syndrome	Gabriele-de-Vries syndrome
**Comorbid disorders family history**	Nil	ID (III-8), ASD (IV-4),
		Seizure/ASD (IV-5),
		ID (IV-7)
**Affected siblings**	Nil	Nil
**Family History of NDD illness**	Nil	Positive
**Disease Symptoms**	Microcephaly	Intellectual impairment
	Intellectual impairment	Facial dysmorphism
	Repetitive behaviors ASD	Dental anomalies,
		Retinal degeneration, Psychiatric manifestations Movement disorder
	Cataract	
	Speech/developmental delay	

### Genetic analysis of WES data for the family ES-4 and ES-10

- Results for proband ES-4 VCF file revealed a heterozygous, missense variant NM_015100.4: c.776 C>T (p. Pro259Leu) in chromosome 1 at exon 19 of the *POGZ* gene classified as pathogenic and deleterious by Franklin Genoox classification. **(Tables 3 & 4)** This variant was validated by sanger sequencing (ABI Prism 3130 Genetic Analyzer Applied Biosystems) to generate Ab1 files which were analyzed using Finch TV 1.4 software. Reference sequences of the genes were downloaded from the Ensembl genome browser. Co-segregation analysis of sanger sequence electropherograms revealed it as *denovo* variant in the proband which did not segregate in the family. These findings solved the complex phenotype of proband ES-4 as a case of White-Sutton syndrome.
- ES-10 proband VCF files uploaded to Franklin Genoox online software revealed a heterozygous, non-frameshift variant NM_003403.5: c.141_143delGGA (p. Glu47del) in chromosome 14 at exon 1 of *YY1* gene **(Tables 3 & 4)** for which co-segregation analysis by conventional sanger sequencing for unaffected parents and siblings was performed by ABI Prism 3130 Genetic Analyzer Applied Biosystems to generate Ab1 files which were analyzed using Finch TV 1.4 software. Reference sequences of the genes were downloaded from the Ensembl genome browser. The results characterized this variant as *denovo* in origin due to no segregation in the family. This discovery has confirmed the phenotype of Gabriele-de Vries syndrome in proband ES-10.

**Table 3:** Wes attributes of the probands ES-4 and ES-10.

Patient ID	ES-4	ES-10
**Suspected Disorder**	WHSUS	GADEVS
**Gene**	POGZ	YY1
**Chromosome**	1	14
**Exon**	19	1
**Nucleotide Change**	c.776 C>T	c.141_143delGGA
**Effect on protein**	Pro259Leu	Glu47del
**Variation Type**	Missense	Non-frameshift

**Table 4:** *Insilico* predictions of the pathogenecity of the variants.

Proband ID	Polyphen 2	SIFT	Mutation Taster	Mutation Assessor	FATHMM	DANN
**ES-4**	Deleterious (0.9)	Deleterious (0.001)	Deleterious (1)	Deleterious (3.8)	Deleterious (0.89)	Deleterious (1)
**ES-10**	Deleterious (0.001)	Deleterious (0.002)	Deleterious (1)	Deleterious (3.6)	Deleterious (0.91)	Deleterious (1)

### Functional analysis for pathogenicity of POGZ and YY1 proteins

Protein modelling was carried out to assess the protein characteristics due to pathogenic variants in the proband ES-4 and ES-10 by HOPE and Alphafold2 online tools. Effects of the pathogenic variants on structure and function of mutant POGZ and YY1 proteins were assessed. In proband ES-4, POGZ conformation shows rigid wild type residue that served as backbone of the protein structure while mutant residue is flexible which disturbs the normal conformation might be required at this position. As the mutant residue is located at a highly conserved region, this POGZ mutation is damaging to the protein. The variation in sizes of the wild type and mutant residues might cause bumps in the conformation. YY1 protein model as depicted by HOPE and alphafold 2, a deletion at position 47, causes the removal of Glutamic acid residue (E) and resultant mutant residue is smaller than the wild type that leads to loss of interaction with the conserved region and abnormal neurodevelopmental has been expressed as pathophysiological consequences This deletion affects the stretch of residues located in a very special protein region (SMAD1/SMAD4 complex) that interacts with signalling pathways including physiological processes of cellular proliferation and differentiation. The structural and functional differences in wild type and mutant aminoacid properties can disturb this region. Phenotypically impacting variants play role beyond pathogenicity, which can alter protein function and molecular mechanisms. Very less studies report the significance of computational approaches to determine the non-framshift mutations, which are topic of debate as they can influence the affinity of protein to bind to other domains required during gene expression. Our study illustrate the diversity of implications of non-frameshift *YY1* deletions, in the manifestation of neurodevelopmental syndromes.

## Discussion

**Fig 4:**
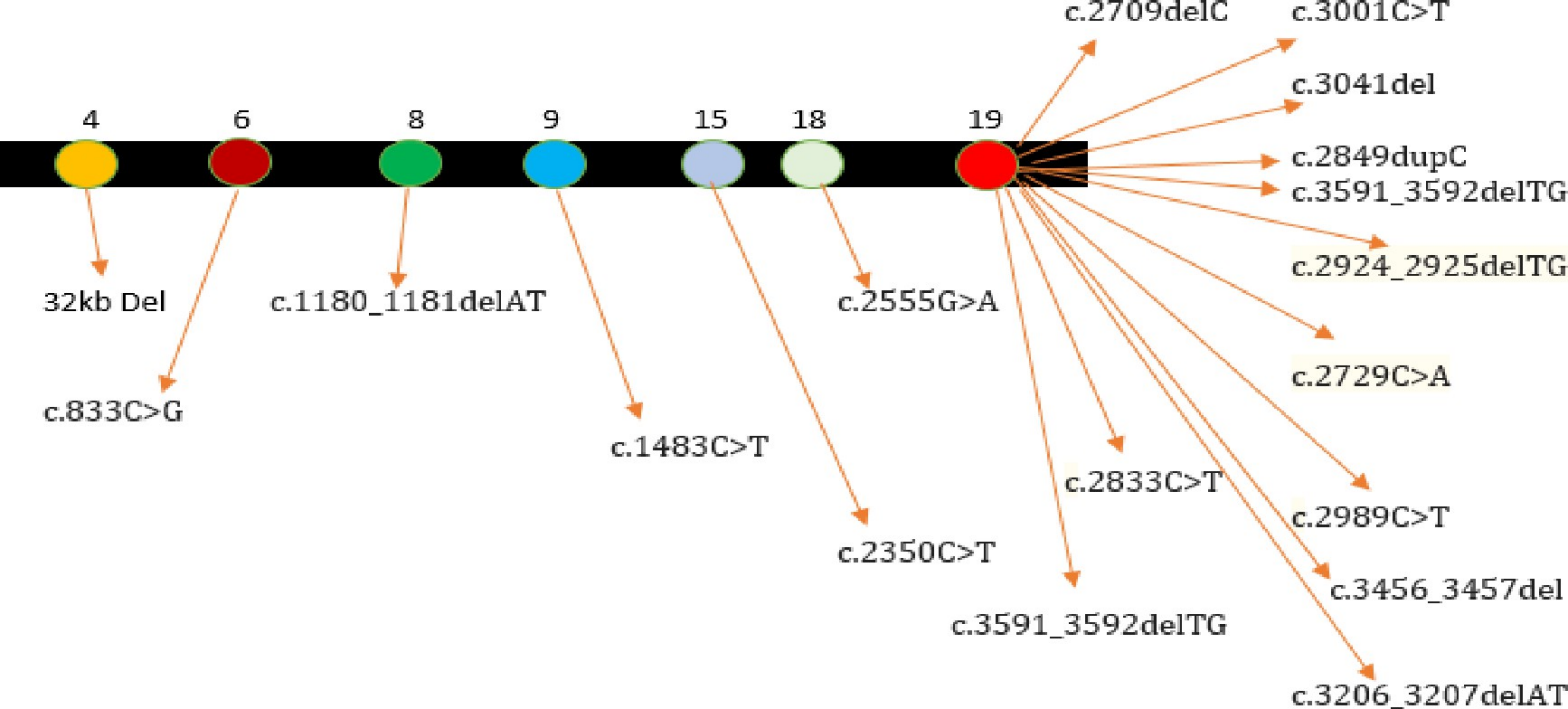
*POGZ* gene showing reported *denovo* loss of function variants at 4,6,8,9,15,18,19 exons, expressed as White Sutton syndrome phenotypes.

We report two unrelated families ES-4 and ES-10, with novel *denovo* variants, a missense variant NM_015100.4: c.776 C>T (p. Pro259Leu) in exons 19 of *POGZ* and a non-frameshift variant NM_003403.5: c.141_143delGGA (p. Glu47del) in exon 1 of *YY1* genes expressed as White Sutton syndromic phenotype in proband ES-4 and Gabriele-de-Vries syndrome manifestation in proband ES-10. *POGZ* gene expresses as multidomain nuclear protein associated with transcriptional control, and its inadequate function has recently been linked to White-Sutton syndrome, a syndromic neurodevelopmental condition, identified in proband ES-4. WHSUS has also been linked to intellectual disability, epilepsy, speech absence or delay, skeletal abnormalities, unusual facial traits, ocular abnormalities, hearing loss, gait abnormalities, obstructive sleep apnea, and gastrointestinal phenotypes, behaviour abnormalities including aggression according to several studies [38] and all this data is in accordance with phenotypic milestones of our study. A high and wide forehead, domed or trapezoidal mouth with downturned edges of the mouth, midface hypoplasia or retrusion, an extended nose base, and an angular nasal arch were the most prevalent attributes [25]. Cortical and cerebellar atrophy, thin corpus callosum, delayed myelination, brainstem hypoplasia, and Dandy-Walker deformity were discovered in brain MRI of patients with POGZ mutations [39]. POGZ gene exons4, 6,8,9,15.18,19 frequently reported [23] with *denovo* variants are depicted in **Fig 4** below.

As great majority of pathogenic variants result in erroneous stop codons, haploinsufficiency has been postulated as an explanation of variant pathogenicity in *POGZ*. The likelihood of loss-of-function sensitivity for this gene indicates the deleterious effects [40]. *POGZ* patients’ disease severity varies greatly, and research addressing genotype-phenotype connection are sparse. POGZ protein enhances chromatin accessibility and expression of clustered synapse genes, and cooccurs loci with *ADNP*, a gene related with autism, according to analyses of *POGZ* knockout mice [25]. *POGZ* deficiency causes a variety of neurodevelopmental diseases, including White-Sutton syndrome. *POGZ* patients have genomic variations, including missense, nonsense, and frameshift variants, as well as deletions. Missense variants appear to be associated with behavioral anomalies rather than intellectual disability, whereas nonsense and frameshift variants are more likely to cause intellectual disability and associated gastrointestinal or urinary tract malformations [23]. Several cases reported so far for WHSUS describe complications in renal, cardiac and neural pathways leading to severe phenotype in these, with about sixteen percent of patients suffered from congenital heart disease [27]. Proband ES-4 in this study showed mild symptoms of heart disease with episodes of tachcardia obseved which is in accordance with previous findings of cases of severe cardiac manifestations. Corneal and retinal pathologies have been reported in WHSUS cases, which strongly support our findings [41, 42]. Behavioural abnormalities have been investigated by multiple research groups hence suggested a strong connection between neurocognitive and neurobehavioural symptoms in WHSUS patients which second our study findings [43]. Proband ES-4 is the first case of neurological pathology involving *POGZ*.

*YY1* gene mutations are expressed as variety of phenotypes with severe neurocognitive domains including Gabriele-de Vries syndrome, a newly described genetic condition that is characterized by developmental delay, craniofacial dysmorphism, intrauterine growth retardation, and various neurologic symptoms [35] which are in accordance with our study findings. This neurodevelopmental condition was caused by a mutation in the *YY1* gene, which encodes the Yin Yang 1 protein, which is important in neuronal development [44]. YY1 is a ubiquitous multifunctional transcription factor that promotes neuregulin-dependent peripheral nerve myelination, inhibits the transcriptional regulators, and promotes the proliferation and survival of neural progenitor cells during the early stages of brain development. Yin and Yang 1 is a transcriptional activator and repressor, first reported in the year 1991 [45], its RNA-binding protein, and 3D chromatin regulator with several functions in neurodevelopmental and maintenance processes [46, 47]. GADEVS, a rare congenital condition characterized by intellectual disability (ID) and multiple physical/behavioral abnormalities, has recently been linked to *YY1* gene caused by either heterozygous sequence variants or deletions involving the entire gene. In this study a denovo deletion is identified in proband ES-10 harboring *YY1* gene and expressed as Gabriele-de Vries syndrome phenotype [48].

**Fig 5:**
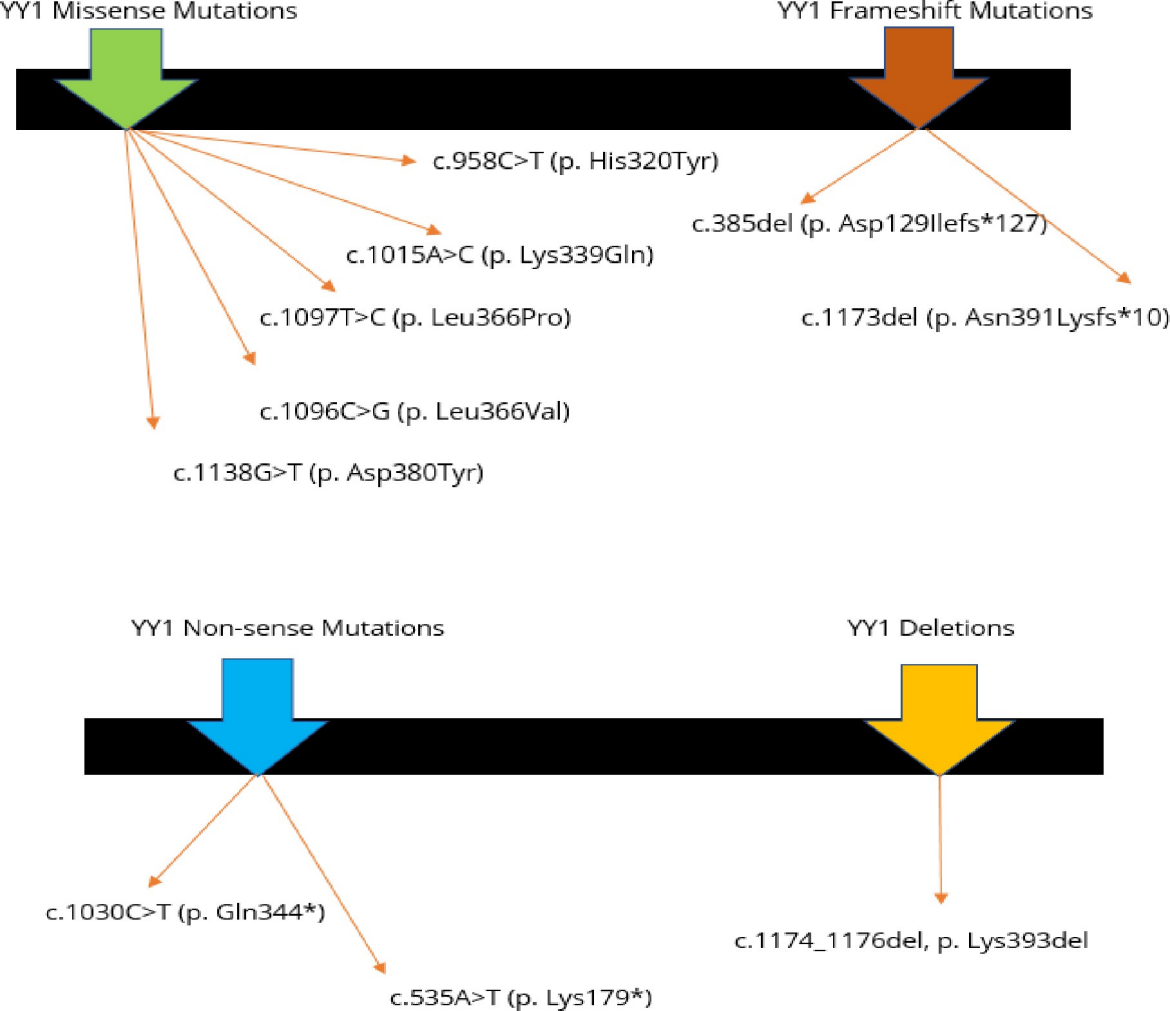
Previously reported *YY1* missense, frameshift, non-sense mutations and deletions in coding regions.

*YY1* was also shown to be implicated in the carcinogenesis of many tumours via p53 downregulation, and it might be used as a marker for therapy, and diagnosis [49]. In addition, *YY1* modulates multiple genes related with neurological developmental disorders, notably *GTF2I*, *KANSL1*, *NRXN2*, *MED12*, *NSD1*, *ZBTB20*, *and HCFC1* [50]. Gabriele-de-Vries genotypes include truncations, deletions and missense variations in the exons of *YY1* gene with a hotspot of mutations in the final exon [51] **(Fig 5)**.

In a recent report of *YY1* gene truncating mutation, depicted phenotypic milestones of learning disability, autoimmune disorder, facial dysmorphism, delayed neuromotor development, indicating that *YY1* defects are variable in multiple cases, majorly these changes were discovered in zinc fingers domain that binds DNA also called repression domain, a few protein truncations but no missense mutation in the N-terminal domain [48]. Our patient from ES-10 family revealed a *denovo* non-frameshift variant NM_003403.5: c.141_143delGGA (p. Glu47del) in chromosome 14 at exon 1 of *YY1* gene not reported before which has added to the genomic data of *YY1* variants and phenotypic characteristics pool. Our study stays unique which not only gets support from previous phenotypes and variants identified but adds to the research domain as comorbidities in GADVES patients include heart, renal, skeletal, eye, teeth, and genital defects in various populations [52].

## Conclusion

We report two probands, ES-4 and ES-10, with two novel *denovo* mutations, a missense variant NM_015100.4: c.776 C>T (p. Pro259Leu) in exons 19 of *POGZ* and non-frameshift variant NM_003403.5: c.141_143delGGA (p. Glu47del) in exon 1 of *YY1* genes causing White Sutton syndrome and Gabriele-de Vries syndrome Respectively. The advent of whole exome sequencing tools has shed light on the previously unidentified neurodevelopmental and intellectual impairment syndromic patients, underpinning of disease genetics formerly assigned to so-called symptomatic causes to aid the clinical research. Expectations are, our study will contribute to the already established protocols for clinical and genetic assessment of rare and unsolved complex phenotypes with comorbid neurological, neuromotor, neuropsychiatric, ophthalmic, auditory, verbal, and behavioral attributes in tertiary care hospitals of Pakistan. It will impact the research spectrum for neurological conditions that mostly arise due to strict ethnic boundaries maintained by the tradition of consanguinity in the culture of our population, especially in the province Punjab. Untiring work done by human genetics research labs will get guidance from this study to explore the domains of White Sutton and Gabriele-de Vries syndrome phenotypes to add to personalized diagnosis and precision medicine investigations.

## Abbreviations

WHSUS: White Sutton syndrome
GADEVS: Gabriele-de-Vries syndrome
ID: Intellectual Disability
IDM: Intellectual Disability with Microcephaly
ASD: Autism spectrum disorder
FATHMM: Functional Analysis Through Hidden Markov Models
SIFT: Scale Invariant Feature Transform
DANN: Dynamic Artificial Neural Network
in: Inches
lb.: Pounds
BMI: Body Mass Index
